# Endoskopische Ohrchirurgie in Deutschland: Umfrage zur aktuellen Situation im internationalen Vergleich

**DOI:** 10.1007/s00106-021-01094-1

**Published:** 2021-08-20

**Authors:** Parwis Agha-Mir-Salim, Miriam Kropp, Alexander Müller

**Affiliations:** Klinik für Hals‑, Nasen-, und Ohrenheilkunde, Kopf- und Halschirurgie, Plastische Operationen, Zentrum für Hörimplantate, Vivantes Hörzentrum Berlin (HZB), Landsberger Allee 49, 10249 Berlin, Deutschland

**Keywords:** Minimalinvasive chirurgische Techniken, Endoskopisches chirurgisches Verfahren, Mittelohr, Erhebung, Operationsdauer, Minimally invasive surgical procedures, Endoscopic surgical procedure, Middle ear, Survey, Operative time

## Abstract

**Hintergrund:**

International hat sich die endoskopische Ohrchirurgie („endoscopic ear surgery“, EES) fest etabliert. In Deutschland wird sie kontrovers diskutiert und unterschiedlich angewendet. Daher erfolgte eine Umfrage zu Angebot, Indikationen, Kontraindikationen und zum zukünftigen Stellenwert der EES.

**Methodik:**

An 141 deutsche Universitäts- und Hauptabteilungen für HNO-Heilkunde, Kopf- und Halschirurgie wurde ein Fragebogen mit 20 Fragen versendet. Die Ergebnisse wurden anhand aktueller Literatur gemäß Suche in PubMed und Google Scholar erörtert.

**Ergebnisse:**

Der Umfragerücklauf betrug 32 % (45 Kliniken). Die EES meist flankierend durchzuführen, gaben 27 Kliniken (60 % der Antwortenden) an. Nur eine Klinik führte alle Ohreingriffe ausschließlich endoskopisch durch. Bei Auftreten intraoperativer Blutungen, Bohrarbeiten am Mastoid oder bei Notwendigkeit bimanuellen Arbeitens wurde zur mikroskopischen Technik („microscopic ear surgery“, MES) gewechselt. Als häufigste Indikationen für die EES wurden Tympanoskopie, Cholesteatom, Retraktionstasche, Eingriffe am Trommelfell und am Gehörgang angegeben. Der Aufwand bei der EES wurde in rund 50 % aller Antworten höher als in der MES eingeschätzt. Bei den EES-Kliniken dominierte mit 78 % der Tragusknorpel als rekonstruktives Transplantat. Nur 4 von 45 antwortenden Kliniken schätzten den zukünftigen Stellenwert der EES in Deutschland als hoch ein.

**Schlussfolgerung:**

Die EES wird in Deutschland zwar eingesetzt, jedoch nur in wenigen HNO-Kliniken in größerem Umfang angewendet. Als problematisch gelten das einhändige Arbeiten, die Durchführung von Bohrarbeiten, Beherrschung von Blutungen und der insgesamt als höher eingeschätzte Aufwand. Häufig wird deshalb die EES flankierend am Ohr angewendet und zwischen EES und MES gewechselt.

## Abstract (en)

**Background:**

Endoscopic ear surgery (EES) has become firmly established internationally. In Germany this technology is controversially discussed and used very differently. Therefore, a survey was carried out on the availability, indications, contraindications, and future significance of EES.

**Methods:**

A questionnaire with 20 questions was sent to 141 German university and central departments of otorhinolaryngology, head and neck surgery. The results are discussed on the basis of current literature retrieved in a search of PubMed and Google Scholar.

**Results:**

The response rate was 32% (45 clinics). Of these respondents, 27 clinics (60%) stated that EES was usually carried out as an accompanying measure. Only one clinic performed ear surgery endoscopically alone. In cases of intraoperative bleeding, mastoid drilling, or if bimanual work was required, the surgeons switched to microscopic ear surgery (MES). The most common indications for EES were tympanoscopy, cholesteatoma, retraction pockets, and interventions on the tympanic membrane and external auditory canal. The necessary resources for EES were estimated to be higher than for MES in 49–50% of the responding clinics. Tragus cartilage dominated as the graft material for reconstruction in EES clinics, at 78%. Only 4 of 45 responding clinics rated the future significance of ESS in Germany as high.

**Conclusion:**

Although EES is used in Germany, only few ENT clinics use it extensively. Reservations relate to one-handed working, drilling, management of bleeding, and the expected high resource demand. Thus, EES in Germany is often performed as an accompanying procedure, with switching between EES and MES.

Der Stellenwert endoskopischer Mittelohreingriffe („endoscopic ear surgery“, EES) wird von vielen Operateur*innen in Deutschland nach wie vor kontrovers diskutiert. Hier ist das Meinungsspektrum breit und reicht von „unnötig“ bis „unentbehrlich“. Zur genaueren Beurteilung des derzeitigen Stands in Deutschland erfolgte eine Umfrage an 141 HNO-Hauptabteilungen. Das Ziel war es, unter Verwendung eines Fragebogens ein möglichst repräsentatives Bild zum tatsächlichen Einsatz der EES-Technik in Deutschland zu erlangen. Hierbei spielten allgemeine, aber auch methodische Fragen eine Rolle.

## Hintergrund

Die bimanuelle, mikroskopische Ohrchirurgie („microscopic ear surgery“, MES) ist ein fest integrierter Bestandteil der HNO-Heilkunde in Deutschland und wird besonders zur Behandlung chronischer Entzündungen und Rekonstruktion des Mittelohrs angewandt [30]. Diese Methode ist bis zum heutigen Zeitpunkt hochspezialisiert und erfordert eine umfangreiche operative Ausbildung. Viele Vorgehensweisen und verwendete Rekonstruktionsmaterialien sind standardisiert. Bei zunehmender persönlicher chirurgischer Erfahrung erlangt die MES eine hohe Individualisierung und wird sowohl in darauf spezialisierten Kliniken als auch in ambulanten Operationszentren und Belegabteilungen angeboten. Dabei ist fast allen Ohrchirurg*innen klar, dass die mikroskopische Technik Schwächen bezüglich der Visualisierung bestimmter Räume, wie beispielsweise des Pro- oder Retrotympanons, aufweist. Daher hat in den letzten 20 Jahren auch bei der Durchführung von Mittelohroperationen die Verwendung des Endoskops (EES-Technik, „endoscopic ear surgery“) zunehmend an Bedeutung gewonnen [3, 34–36].

Endoskopische Techniken sind in Deutschland fest etablierte Bestandteile der chirurgischen Therapie. Hierdurch steht die apparative Ausrüstung den meisten HNO-Kliniken problemlos zur Verfügung. Der Einstieg in diese Technik geht bei gleichen Zielen durchaus mit einer Veränderung des chirurgischen Vorgehens einher. Bedingt wird dieses durch die intraoperative Einhändigkeit, die visuelle Kontrolle des Operationsfelds mit einem Monitor und die optischen Eigenschaften des Endoskops, wie dem zweidimensionalen Weitwinkelblick [6]. Bei Unerfahrenen sind typische Komplikationen, wie beispielsweise die Verletzung eines freiliegenden N. facialis oder des ossikulären Mittelohrsystems, durchaus bekannt [38]. Allerdings kann auch erfahrenen Operateur*innen der Umstieg auf das Endoskop im Mittelohr schwerfallen. Meist gelingt jedoch eine Umgewöhnung bereits nach wenigen Eingriffen [13]. Mit zunehmender Anzahl durchgeführter EES-Eingriffe gewinnt diese Methode für Operierende meist an Nutzen.

Allein in den letzten 12 Monaten sind 466 Artikel zur EES publiziert worden, was den aktuellen Stellenwert im internationalen Vergleich widerspiegelt. In Deutschland wurden die Indikationen und Vorteile dieser Technik von Preyer [26] ausführlich beschrieben und anhand der aktuellen nationalen und internationalen Literatur analysiert. Zum damaligen Zeitpunkt wurde festgestellt, dass sich die EES-Technik in Deutschland noch nicht als Routineverfahren etablieren konnte und in den Kinderschuhen stecke.

Es war somit von Interesse, welchen Stellenwert die EES in Deutschland 5 Jahre später erreicht hat. Dies sollte trotz der Komplexität und Problematik [33] mit einer Umfrage erfasst werden. Der selbst formulierte Fragenkatalog orientierte sich an der bisher einzigen Voruntersuchung [10]. Diese Ergebnisse werden unter Betrachtung aktueller nationaler und internationaler Publikationen diskutiert.

Die Umfrage umfasste,

- ob EES-Eingriffe angeboten werden,
- wie hoch der prozentuale Anteil von EES-Eingriffen bezogen auf alle Mittelohreingriffe ist,
- welche Instrumente verwendet werden,
- die Einschätzung des Gesamtaufwands im Vergleich zur MES,
- die Indikationen und Kontraindikationen der EES-Technik,
- welche Transplantate verwendet werden,
- die Vor- und Nachteile der EES-Technik,
- die Einschätzung des zukünftigen Stellenwerts.

Alle den Autoren zur Verfügung stehenden Kontaktdaten von deutschen HNO-Kliniken wurden verwendet. Zur Diskussion der eigenen Ergebnisse wurden v. a. die in PubMed (National Library of Medicine) und Google Scholar (Google LLC) erschienenen Publikationen der letzten 12 Monate berücksichtigt.

## Methodik

### Studienpopulation

Es wurden 141 Fragebögen persönlich elektronisch an Universitäts- und Hauptabteilungen für Hals‑, Nasen‑, Ohrenheilkunde, Kopf- und Halschirurgie in Deutschland adressiert. Hiervon waren 32 universitäre und 109 nichtuniversitäre Einrichtungen. Insgesamt antworteten 45 der Angeschriebenen. Darunter waren 10 Universitäts- und 35 nichtuniversitäre Kliniken. Die Nachfrage bei der Deutschen Gesellschaft für Hals-Nasen-Ohren-Heilkunde, Kopf- und Hals-Chirurgie (DGHNO-KHC) ergab, dass derzeit 38 Universitätskliniken und 128 nichtuniversitäre HNO-Hauptabteilungen gelistet sind. Unter der Annahme, dass dies der tatsächlich vorhandenen Anzahl entspricht, wurden somit 85 % der HNO-Kliniken in Deutschland eingeschlossen.

### Messinstrument

Der Fragebogen wurde in Microsoft Word® (Fa. Microsoft, Redmond/WA, USA) erstellt und aktive Checkboxen zum Ankreuzen der Antworten eingefügt. Er bestand aus 20 Fragen unterschiedlichen Designs. Davon waren 19 geschlossen, eine halboffen. Bei 9 Fragen stand eine Einfachauswahl zur Verfügung, bei 11 eine Mehrfachauswahl. Folgendes war zu beantworten:

1. Führen Sie endoskopische Operationen am Mittelohr aus? (ja, nein)
2. Wenn nein, warum nicht? (kein Nutzen; kein*e Operateur*in; Interesse, aber keine Kapazität; zu aufwendig)
3. Wenn ja, wie viele Eingriffe bezogen auf die Gesamtanzahl/Jahr? (< 10, 10–25, 25–50, > 50 %)
4. Welche Indikation sehen Sie als relevant an? (Gehörgangspathologie, Trommelfellrekonstruktion, Ossikuloplastik, Retraktionstasche, Cholesteatom, Stapesplastik, Tympanoskopie [Mehrfachantwort möglich])
5. Welche Kontraindikationen sehen Sie? (enger Gehörgang, Bohrarbeiten, Ossikuloplastik, Blutungsneigung, Kinder [Mehrfachantwort möglich])
6. Wie viele Operateur*innen? (1, > 1)
7. Führen Sie bei der EES eine Gehörgangsinzision durch? (ja, nein, manchmal)
8. Welches Instrumentarium verwenden Sie? (Sauginstrumente, konventionelle Instrumente, gewinkelte Instrumente, für die EES: Bohrer, Piezo, monopolare Nadel)
9. Welche Endoskope verwenden Sie? (Durchmesser: < 3 mm, > 3 mm; Winkel: 0°, 30°, 45°, 70° [Mehrfachantwort möglich])
10. Halten Sie bei der EES das Mikroskop steril bezogen bereit? (ja, nein, manchmal)
11. Wenn nein, warum nicht? (nicht erforderlich, zu hoher Zeit- und Kostenaufwand)
12. Wenn ja, welche Indikationen sehen Sie für einen „switch“ zur mikroskopischen Technik? (Blutung, bimanuelles Arbeiten erforderlich, umfangreiche Bohrarbeiten am Mastoid, Bogengangsfistel, freiliegender N. facialis, Otoliquorrhö [Mehrfachantwort möglich])
13. Schätzen das Verhältnis Mikroskop‑/Endoskopeinsatz an Ihrer Klinik prozentual ein. (Mikroskop allein, Endoskop nur flankierend, > 50 % Mikroskop, > 50 % Endoskop, „switch“ Mikroskop bei Mastoidarbeiten, nur Endoskop [Mehrfachantwort möglich])
14. Welche Bereiche sind Ihrer Einschätzung nach besonders für den Endoskopeinsatz am Ohr geeignet? (Trommelfell, Gehörgang, rundes Fenster, ovales Fenster, Sinus tympani, Epitympanon, Antrum, Protympanon, Mastoid [Mehrfachantwort möglich])
15. Welche Bereiche sind besonders geeignet für den Mikroskopeinsatz? (Trommelfell, Gehörgang, rundes Fenster, ovales Fenster, Sinus tympani, Epitympanon, Antrum, Protympanon, Mastoid [Mehrfachantwort möglich])
16. Schätzen Sie den Gesamtaufwand endoskopischer Mittelohreingriffe im Vergleich zur mikroskopischen Technik ein. (geringer, identisch, höher, abhängig von der „Lernkurve“ des gesamten Teams)
17. Wo heben Sie bei endoskopischen Ohroperationen benötigte Transplantate? (Tragus, Concha, Cymba, Temporalisfaszie, gar nicht wegen Verwendung eines Xenografts)
18. Was ist der größte Nachteil der EES gegenüber der mikroskopischen Technik? („single-handed“, schlechte Übersicht bei stärkerer Blutung, längere Op.-Dauer, Operation über Monitor und Endoskopkamera, kein binokularer räumlicher Blick, andere [Mehrfachantworten und freie Antwort möglich])
19. Was ist der größte Vorteil der EES gegenüber der mikroskopischen Technik? (keine Inzision, weniger Schmerzen postoperativ, geringerer intra- und postoperativer Aufwand, kostengünstiger, kürzere Op.-Dauer, seltener Komplikationen [Mehrfachantwort möglich])
20. Wie schätzen Sie perspektivisch den zukünftigen Stellenwert der EES ein? (sehr hoch, hoch, mittel, gering, kein, unklar)

### Auswertung

Die Auswertung und graphische Darstellung einzelner Ergebnisse erfolgte in Microsoft Excel® (Microsoft, Redmond US-WA).

Die 18 Publikationen aus der Literatursuche, die als Review konzipiert waren, wurden zur Diskussion der eigenen Ergebnisse herangezogen.

## Ergebnisse

Von den 141 versendeten Fragebögen wurden 45 Fragebögen (Responserate von 32 %) beantwortet zurückgeschickt. Hiervon waren 10 Universitätskliniken und 35 nichtuniversitäre Hauptabteilungen.

### Grundsätzliche Fragen zur EES

#### Angebot und Operateur*in.

Von 27 der 45 Kliniken (60 %) wurde angegeben, dass sie die EES-Technik verwenden (im Folgenden EES-Klinik genannt). Die übrigen 18 Kliniken (40 %) gaben an, keine EES anzubieten (im Folgenden MES-Klinik genannt). Von denselben Kliniken bestand in mehr als 50 % durchaus Interesse an EES, die Technik wurde jedoch aufgrund eines zu hohen Aufwands und mangelnder Kapazitäten nicht eingeführt. Als Begründung gaben 6 Kliniken (33 %) an, dass sie keinen ersichtlichen Nutzen in der EES erkennen könnten. In jeweils einer Klinik war kein*e Operateur*in vorhanden, oder es wurde die Anschaffung von Sauginstrumenten mangels zertifizierter Sterilisierbarkeit durch die Zentralsterilisation abgelehnt.

#### Anteil EES-Operationen und Operateur*in.

In 70 % der EES-Kliniken erfolgen maximal 20 % aller Ohreingriffe pro Jahr endoskopisch. In 22 % der Fälle führen diese Kliniken zwischen 25 und 50 % und in 7 % mehr als 50 % dieser Operationen jährlich mit der EES-Technik durch. Es wurde weiterhin erfragt, wie viele Ohroperateur*innen an den Kliniken zur Verfügung stehen. Von den EES-Anwendenden gaben 26 % an, dass sie nur eine*n Operateur*in zur Verfügung hätten, in 70 % wurde die Anzahl der Operateur*innen > 1 angegeben. Die übrigen 4 % machten keine Angabe.

### Indikation und Durchführung sowie Vergleich von EES und MES

Die Fragen zur praktischen Durchführung der EES wurden von 43 Kliniken beantwortet und damit sowohl von EES- als auch MES-Kliniken. Bei einigen methodischen Fragen traten erhebliche Unterschiede in den Antworten auf, weshalb die Ergebnisse in diesen Fällen vergleichend zwischen EES- und MES-Kliniken analysiert wurden.

#### Indikationen und Kontraindikationen (Mehrfachantwort möglich).

**Figure 1:**
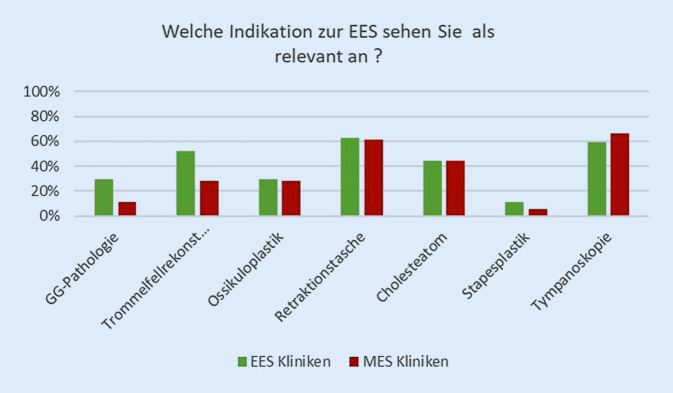

Als häufigste Indikationen für die Anwendung der EES wurden in 63 % der Fälle eine Retraktionstasche und in 59 % die Tympanoskopie angegeben. Von den EES-Anwendenden wurde im Vergleich zu ausschließlichen MES-Kliniken zusätzlich in 52 % der Fälle der Einsatz zur Trommelfellrekonstruktion und in 30 % für Eingriffe am Gehörgang gesehen. Die Stapesplastik wurde mit 11 % der EES und 5 % der übrigen Kliniken nur selten als Indikation gesehen (Abb. 1).

Insgesamt wurden ein zu enger Gehörgang, Bohrarbeiten und Blutungsneigung von über 60 % der Kliniken als Kontraindikation angesehen.

#### Operativer Zugangsweg.

Die Häufigkeit der Durchführung einer Gehörgangsinzision als Zugangsweg wurde von EES-Kliniken in 41 % mit „stets“, in 37 % mit „manchmal“ und in 22 % mit „nie“ angegeben.

#### Instrumentarium.

In allen Kliniken kommen konventionelle und in 59 % zusätzlich abgewinkelte Ausrüstung ohne Saugkanal zum Einsatz. Bezüglich des verwendeten Instrumentariums wurde von den meisten EES-Kliniken Op.-Material mit Saugkanal (74 %) benannt. Bohrer (33 %), Piezotechnik (7 %) oder eine monopolare Nadel (4 %) wurden seltener verwendet.

#### Transplantate (Mehrfachantworten möglich).

In 56 % aller antwortenden Kliniken wurden Tragusknorpel und Perichondrium als Transplantate benannt, gefolgt von Temporalisfaszie (29 %) und Conchaknorpel (22 %). Bei EES-Anwendenden wurde in 78 % ausschließlich Tragusmaterial, in 41 % zusätzlich oder allein Temporalisfaszie und in 30 % Conchaknorpel verwendet. Cymbaknorpel wurde insgesamt mit 7 % seltener eingesetzt. Xenografts kamen überhaupt nicht zum Einsatz.

#### Endoskope.

Von den EES-Kliniken verwendeten 70 % Endoskope mit einem geringeren Durchmesser als 3 mm. Jeweils in 74 % der Kliniken wurden 0°- und 30°-Optiken eingesetzt, 48 % verwendeten zusätzlich 45°- und 30 % 70°-Endoskope. Die 3‑D-Technik wurde von keiner Klinik angewandt.

#### Bereithalten des Mikroskops bei der EES.

Das Mikroskop hielten 66 % der EES-Kliniken stets steril bezogen bereit, je 15 % „nie“ oder nur „manchmal“, da dies als nicht erforderlich angesehen wurde.

#### Gründe zum Wechsel zwischen MES- und EES-Technik.

Einen Wechsel zur mikroskopischen Technik sahen EES-Kliniken v. a. bei umfangreicheren Bohrarbeiten am Mastoid (93 %), bei der Notwendigkeit von bimanuellem Arbeiten (81 %) oder bei Blutungen (67 %) als erforderlich an. Seltener wurde dies bei einer Bogengangsfistel (52 %), einer Otoliquorrhö (52 %) oder einem freiliegenden N. facialis (26 %) gesehen.

#### Anteil MES/EES.

Die Antworten der 3 Kernfragen zum Angebot endoskopischer Ohreingriffe, dem Verhältnis Mikro‑/Endoskopeinsatz und der perspektivische zukünftige Stellenwert wurden bezogen auf Universitätskliniken und nichtuniversitäre Kliniken gegenübergestellt. Prozentual bieten annähernd alle Universitätskliniken (90 %) gegenüber nur 51 % der nichtuniversitären Kliniken EES an. In beiden Bereichen wurde das Endoskop meist nur flankierend eingesetzt. An Universitäten werden in 80 %, an nichtuniversitären Kliniken in 26 % mehr als die Hälfte der Operationen mikroskopisch durchgeführt. Nichtuniversitäre Kliniken führen jedoch mit 26 % der Antwortenden deutlich häufiger ausschließlich mikroskopisch gestützte, im Fall einer Klinik ausschließlich, endoskopische Mittelohreingriffe durch (Tab. 1).

**Table 1:** 

		Universitätsklinik (*n* = 10; Anteil in %)	Nichtuniversitäre Klinik (*n* = 35; Anteil in %)
Durchführung endoskopischer Operationen am Mittelohr		90	51
Verhältnis Mikroskop‑/Endoskopeinsatz (Mehrfachantworten waren möglich)	Endoskop nur flankierend	60	46
	> 50 % Mikroskop	80	26
	Nur Mikroskop	10	26
	Nur Endoskop	0	3
Perspektivischer Stellenwert der EES	Sehr hoch	0	11
	Hoch	0	3
	Mittel	70	49
	Gering	10	23
	Gar nicht	0	3
	Unklar	20	14

**Figure 2:**
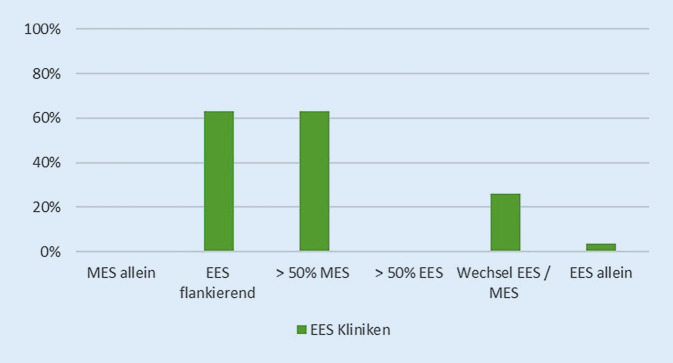

Bei ausschließlicher Betrachtung der EES-Anwendenden wurde in 63 % der Fälle nur flankierend endoskopisch operiert und mehr als die Hälfte der Operationen mikroskopisch durchgeführt. Ein erforderlicher Wechsel von der Endoskopie zur Mikroskopie erfolgte in 26 % der Fälle. Eine EES-Klinik gab an, ausschließlich endoskopisch zu operieren (Abb. 2).

#### Besonders geeignete Bereiche für den Einsatz der EES.

**Figure 3:**
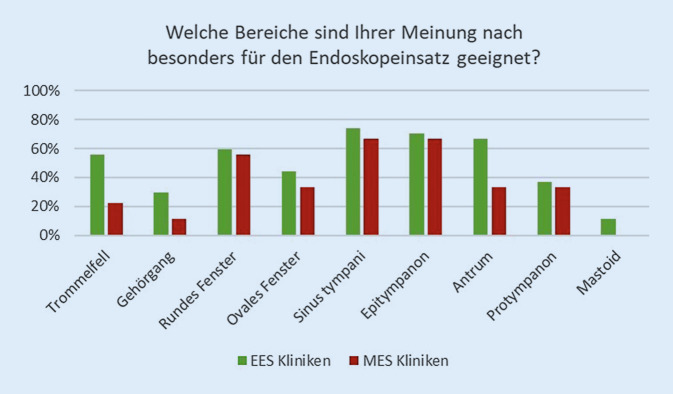

Bezogen auf alle teilnehmenden Kliniken wurden der Sinus tympani (71 %), das Epitympanon (69 %) und das runde Fenster (58 %) als besonders für das Endoskop geeignete Bereiche angesehen. Die EES-Kliniken sahen zusätzlich häufiger eine Indikation zum endoskopischen Operieren am Antrum (67 %), aber v. a. auch am Trommelfell (56 %) und zu 30 % am Gehörgang (Abb. 3).

Die mikroskopische Technik wurde von MES- und EES-Kliniken gleichermaßen am Mastoid (> 75 %), dem Gehörgang (> 70 %) und am ovalen Fenster (> 65 %) favorisiert.

#### Vergleich Aufwand MES/EES.

Beim Vergleich des Gesamtaufwands der endoskopischen mit der mikroskopischen Technik schätzten alle antwortenden EES-Kliniken diesen bei der EES als höher (49 %), aber auch abhängig von der „Lernkurve“ des Op.-Teams (69 %) ein. Ausschließliche MES-Anwender sahen den Aufwand ebenfalls mit 50 % der Antworten als höher an und in 56 % eine Abhängigkeit von der Lernkurve.

#### Vor- und Nachteile sowie zukünftiger Stellenwert der EES-Technik.

Als größte Vorteile der EES-Technik wurden von den EES-Kliniken das Umgehen einer Gehörgangsinzision (56 %) und die daraus resultierenden geringeren postoperativen Schmerzen (37 %) angegeben.

Als größte Nachteile der EES-Technik wurden von den EES-Kliniken das einhändige Operieren (89 %), eine schlechtere Übersicht (78 %), der fehlende binokulare Blick (41 %) und längere Op.-Dauern (37 %) angegeben. Eine Klinik empfand die Notwendigkeit der langen Stabilisierung des Endoskops mit der Hand bei aufwendigen Operationen als belastend. Explizit wurden in einem anderen Fall umfangreichere endoskopische Bohrarbeiten wegen der schlechteren Übersicht und möglicher Schäden des Endoskops durch den Bohrer als ungünstig angesehen.

**Figure 4:**
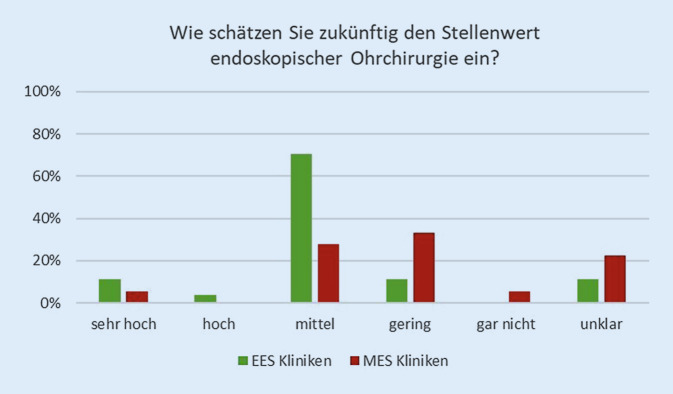

Der Fragenkatalog wurde mit einer Einschätzung zum zukünftigen Stellenwert der EES-Technik anhand einer 6‑stufigen Skala abgeschlossen. MES-Kliniken schätzten diesen mehrheitlich als „gering“ (33 %), „mittel“ (28 %) bzw. „unklar“ (22 %) ein. EES-Kliniken hingegen sahen in 70 % der Fälle den Stellenwert als „mittel“ bzw. „gering“ und nur in 11 % als unklar an. Lediglich 4 EES- und eine MES-Kliniken schätzten den zukünftigen Stellenwert der EES-Technik als „sehr hoch“ ein (Abb. 4).

Im internationalen Vergleich entstand bei dieser Umfrage der Eindruck, dass die EES in Deutschland immer noch zurückhaltend eingesetzt wird. Zusätzlich speziell für die EES entwickelte Op.-Instrumente sowie chirurgisches Training könnten den Stellenwert dieser Technik in Deutschland erhöhen und sie in einem internationalen Vergleich zu einem berechtigten festen Bestandteil der ohrchirurgischen Ausbildung machen.

## Diskussion

Endoskopische Ohrchirurgie wurde im deutschsprachigen Raum zuletzt und einzig von Preyer 2016 detailliert beschrieben [26]. Trotz der hohen internationalen Präsenz dieses Themas ist für Deutschland 5 Jahre später nicht klar, welchen Stellenwert diese Technik in der mikroskopisch geprägten Ohrchirurgie tatsächlich spielt. Im internationalen Vergleich unter Betrachtung der allein in den vergangenen 12 Monaten erschienen 466 Publikationen (hiervon 18 Reviews) wird der subjektive Eindruck verstärkt, dass die EES-Technik in Deutschland weiterhin eher wenig Beachtung findet. In mehreren aktuellen Übersichtsarbeiten bekannter Arbeitsgruppen aus den USA, Italien, den Vereinigten Arabischen Emiraten, Brasilien und der Türkei wurden hingegen der Stellenwert, die chirurgische Anatomie und die Indikationen für die EES-Technik klar formuliert. Übereinstimmend wurde dort festgestellt, dass die endoskopische Technik dem Mikroskop überlegen ist, wenn die Präparation ein weiteres Gesichtsfeld erfordert oder in mikroskopisch schlecht einsehbaren Gebieten gearbeitet werden muss. Außerdem wird betont, dass jede otologische Operation grundsätzlich endoskopisch durchgeführt werden könnte. Als logische Konsequenz wird die Entwicklung neuer Instrumente sowie eine einheitliche Nomenklatur und Standardisierung der Technik gefordert [6, 8, 11].

Die einzige Arbeit, in der mittels einer Umfrage der Einsatz der EES-Technik schon näher untersucht wurde, stammt von Kapadiya und Tarabichi aus dem Jahr 2018 [10]. Hier wurden 385 Mitglieder der American Academy of Otolaryngology – Head and Neck Surgery, welche sich als Otolog*in registriert hatten, über eine Online-Plattform in den Jahren 2010 und 2018 angeschrieben. Die Autoren formulierten 13 Fragen zu Indikationen und Einsatzgebieten der EES sowie dem Alter der Operateur*innen und der Erfahrung in anderen endoskopischen Techniken. Der Rücklauf der Fragebögen betrug 47 (12 %) im Jahr 2010 und 28 (7 %) im Jahr 2018. Die Verfasser stellten fest, dass über die Zeit das endoskopische Vorgehen zunehmend bei Cholesteatomen und flankierend zur mikroskopischen Technik favorisiert wurde. Es wurde auch gezeigt, dass die Anzahl der Antwortenden, die einen speziellen Operationskurs besucht hatten, deutlich anstieg. Während 2010 noch 81 % angaben, keinen entsprechenden Kurs besucht zu haben, lag diese Zahl 2018 nur noch bei 14 %.

Die eigene Studie sollte deskriptiv die Situation in Deutschland hinsichtlich der Anwendung der EES-Technik erfassen. Voruntersuchungen in dieser Form lagen für den deutschsprachigen Raum bisher nicht vor, weshalb die Fragen anhand der eigenen Erfahrungen und in Anlehnung an die Publikation von Kapadiya und Tarabichi [10] formuliert wurden. Daher war weder eine Validierung noch eine detaillierte statistische Aufarbeitung möglich.

Wichtig für die Beurteilung der Repräsentativität von Umfragen sind die Antwortrate, aber auch die Zusammensetzung der Stichprobe [33]. Die Fragebögen wurden auf ärztlicher Ebene versandt mit dem Ziel, die Responserate durch diese persönliche Kontaktaufnahme zu erhöhen. Von den übrigen Kliniken lagen keine persönlichen Kontakte vor. In diesen Fällen wäre eine offizielle E‑Mail-Adresse einer Klinik angeschrieben worden, ohne dass klar gewesen wäre, wer tatsächlich die Umfrage beantworten würde. Die Autor*innen haben deshalb diese nicht eingeschlossen. Es sollte weiterhin eine kurzfristige Momentaufnahme erfolgen. Der Rücklauf der Antwortenden innerhalb der ersten Woche nach dem elektronischen Versand lag bei 86 %, was somit diesem Ziel durchaus entsprach.

Grundsätzlich ist bei einer Responserate von weniger als 100 % stets zu hinterfragen, ob die Stichprobe tatsächlich repräsentativ war. Man kann hierzu feststellen, dass 85 % der Kliniken in Deutschland elektronisch erreicht werden konnten und hiervon knapp ein Drittel geantwortet hat. Die Responserate von 32 % erscheint vor diesem Hintergrund zunächst zwar nicht all zu hoch und sollte nach Möglichkeit in einem solchen „setting“ mindestens 40 % betragen [33]. Bei Betrachtung anderer Onlineumfragen in der Hals-Nasen-Ohren-Heilkunde in Deutschland entspricht allerdings die vorliegende Antwortrate durchaus den dort erzielten Werten zwischen 30 und 40 % [12, 27]. Bezogen auf die einzige vergleichbare Studie [10] war die Responserate sogar deutlich höher.

Auf Nachfrage beim größten Hersteller von Sauginstrumenten für EES wurden mehr als 50 Kliniken in Deutschland beliefert. Die Aufbereitung dieser Instrumente ist im Übrigen nach DIN EN ISO 17665-1–3 anerkannt in einem unabhängigen Prüflabor validiert [31]. Ausgehend von der Gesamtzahl der in der DGHNO-KHC bekannten 166 HNO-Hauptabteilungen in Deutschland entspräche der Anteil von EES-Kliniken 33 %. In der Annahme, dass die mit Sauginstrumenten versorgten Kliniken in Deutschland diese auch tatsächlich anwenden, entsprächen die antwortenden 27 EES-Kliniken der Umfrage einem Anteil von 54 % der Belieferten. Trotz dieser Unwägbarkeiten lässt sich somit feststellen, dass mindestens die Hälfte der EES-Kliniken in Deutschland auf die Umfrage geantwortet haben müssten. Bezogen auf alle Responder haben EES-Anwendende nicht wesentlich häufiger geantwortet. Auch das Verhältnis kontaktierter universitärer zu nichtuniversitären Kliniken sollte zur Beurteilung der Repräsentativität betrachtet werden. Sowohl in der Stichprobe als auch bei den Antwortenden war dies dem Gesamtverhältnis entsprechend. Aufgrund all dieser Gesichtspunkte kann somit davon ausgegangen werden, dass das Kriterium einer weitestgehenden Repräsentativität der Antwortenden erfüllt sein sollte.

### Grundsätzliche Fragen zur EES-Technik

#### Angebot und Operateur*in.

Insgesamt war die hohe Anzahl reiner MES-Kliniken überraschend. Von besonderem Interesse waren die Gründe, aus denen Kliniken EES nicht in das Behandlungsangebot aufnahmen. Angegeben wurde hier ein zu hoher Aufwand, mangelnde Kapazitäten oder ein nicht erkennbarer Nutzen der Methode. Es ist zu diskutieren, ob die anfänglich längeren Op.-Dauern am ehesten zur Ablehnung dieser Technik geführt haben müsste. Es existieren gerade hierzu eine Reihe von Publikationen, die nach der Startphase einen durchaus vergleichbaren Aufwand beider Methoden belegen, womit dies anfangs sicher zu erwarten, nicht aber als dauerhaft anzusehen wäre [5, 29]. Es wurde in früheren Untersuchungen gezeigt, dass zur Vermeidung längerer Op.-Dauern v. a. in der Anfangsphase zur Einführung der EES in einer Klinik das „setting“ und der Ablauf klar strukturiert werden sollten [5, 11]. An einer Studie von 8 erfahrenen Ohrchirurg*innen konnten Lucidi und Presutti am Beispiel der Typ-I-Tympanoplastik zeigen, dass besonders die ersten 5 Eingriffe langwierig waren. Nach etwa 30 Eingriffen kam es zu einer weiteren deutlichen Absenkung der Op.-Dauern [13].

Die Einführung der EES-Technik ist selbstverständlich eine individuelle und grundsätzliche Entscheidung jeder einzelnen Klinik. Eine Ablehnung dieser Methode aus den genannten Gründen jedoch erscheint unter Betrachtung vorliegender Literatur schwer nachvollziehbar. Eine vergleichende Untersuchung zwischen EES und MES beispielsweise hinsichtlich der Schnitt-Naht-Zeiten oder des materiellen Ressourcenverbrauchs für Deutschland wären hilfreich, diese Einschätzung zu objektivieren.

#### Anteil EES-Operationen und Operateur*in.

Bezüglich der Eingriffshäufigkeit fanden in den meisten EES-Kliniken endoskopische Eingriffe eher selten statt. Lediglich etwas mehr als ein Viertel der EES-Kliniken verwendete die EES-Technik bei mehr als 25 % aller Mittelohreingriffe. Nur 2 EES-Kliniken führten in Deutschland jährlich mehr als 50 % der Mittelohroperationen endoskopisch durch. Hieraus ist ableitbar, dass diese Technik offensichtlich an einer Reihe von Kliniken implementiert ist, aber insgesamt nur von wenigen Operateur*innen und i. d. R. nur selten angewendet wird. Werden universitäre und nichtuniversitäre Einrichtungen verglichen, so ist die EES in annähernd allen Universitätskliniken, aber nur in der Hälfte der übrigen Kliniken etabliert. Unabhängig hiervon wurde in allen die EES anbietenden Kliniken diese Technik von ein oder 2 Operateur*innen durchgeführt. Hier liegt die Einschätzung nahe, dass in den meisten antwortenden Kliniken die EES viel häufiger von Universitätskliniken angeboten wird, diese Technik aber insgesamt eher als komplementäres Angebot zur MES von wenigen Operateur*innen durchgeführt wird. Die einzige EES-Klinik, die alle Mittelohreingriffe endoskopisch durchführt, war jedoch keine Universitätsklinik.

### Indikationsstellung und Durchführung der EES sowie Vergleich zur MES

Dieser Fragenbereich wurde sowohl von EES-Kliniken als auch von einzelnen MES-Kliniken beantwortet. Alle Antwortenden sahen annähernd gleiche Indikation zur Anwendung der EES-Technik bei der Tympanoskopie, einem Cholesteatom, am Antrum und bei Retraktionstaschen. Dies entspricht durchaus den Ergebnissen anderer Untersuchungen [17, 19, 25, 32]. Nur selten wurde eine Stapesplastik als Indikation angesehen. Wie in einem Vergleich zwischen endoskopischer und mikroskopischer Technik an knapp 200 Fällen gezeigt wurde, weisen insbesondere die Hörergebnisse sowie die Schnitt-Naht-Zeiten keine signifikanten Differenzen auf [14], weshalb das endoskopische Vorgehen hier außer einem etwas geringeren Aufwand auch keinen wesentlichen Vorteil gegenüber der MES erkennen lässt.

EES-Kliniken sahen wesentlich häufiger als MES-Kliniken eine zusätzliche Indikation für die EES-Technik bei Arbeiten am Gehörgang und am Trommelfell. Mikroskopisch ist die Kontrolle der vorderen Trommelfellabschnitte und des tympanomeatalen Winkels bei vorspringender Gehörgangswand häufig eingeschränkt und nur mittels operativer Gehörgangserweiterung möglich. Endoskopisch ist dies jedoch ohne zusätzliche Intervention zu erreichen, sodass genau dieser Umstand zu diesem Ergebnis geführt haben müsste [25].

Die endoskopische Durchführung einer Operation kann im Vergleich zur MES-Technik bekanntermaßen die Notwendigkeit chirurgischer Zugänge absenken. Mögliche Vorteile könnten v. a. in einer geringeren postoperativen Schmerzentwicklung bestehen. In einer Studie an insgesamt 60 Patienten mit jeweils transmeatal endoskopischer, endauraler Inzision und retroaurikulärem Zugang konnten allerdings keine signifikanten Unterschiede bezüglich dieser Vermutung aufgezeigt werden [4]. Insgesamt kann nach der vorliegenden Umfrage festgestellt werden, dass der chirurgische Zugangsweg in den EES-Kliniken in Deutschland sehr unterschiedlich gehandhabt wird. Eventuell besteht bei der Häufigkeit der Anlage eines endauralen Schnitts ein Zusammenhang mit der Möglichkeit, problemlos ein eventuell notwendiges Transplantat über denselben Schnitt zu heben.

Erwartungsgemäß verwendeten alle EES-Kliniken das vorhandene konventionelle Instrumentarium. Eine stärkere Blutung kann das endoskopische Vorgehen im Ohr erheblich erschweren. Untersuchungen haben jedoch gezeigt, dass diese durch den lokalen Einsatz von Vasokonstringenzien auf kleinen Wattestückchen ausreichend zu kontrollieren sind [1]. Instrumente mit Saugkanal können dabei zusätzlich hilfreich sein. In knapp 3 Viertel der EES-Kliniken kam passend hierzu zusätzlich Material mit Saugkanal bei stärkeren Blutungen zum Einsatz. Die Anschaffung und Aufbereitung von Sauginstrumenten bedeuten allerdings einen höheren Aufwand, weshalb sie anscheinend auch nicht regelhaft verwendet wurden.

Der Bohrereinsatz ist endoskopisch nur sehr eingeschränkt möglich, da die Optik durch die Bohrrückstände und Spülflüssigkeit verschmutzt. Es wurden zwar Einsatzmöglichkeiten am Mastoid mit in die Spülflüssigkeit eingetauchtem Endoskop und Bohrer entwickelt [35], insgesamt ist die Sicht jedoch nicht optimal. Die geringe Häufigkeit des Bohrereinsatzes in nur einem Drittel der EES-Kliniken war daher zu erwarten. Trotzdem bietet die EES-Technik aber gerade angesichts der derzeitigen SARS-CoV-2-Pandemie gegenüber dem mikroskopischen Vorgehen deutliche Vorteile mit einer geringeren Tröpfchenbildung im Op.-Feld [2]. Die Entwicklung eines geeigneten Systems mit Spülung und Absaugung wäre daher auch aus diesem Grund sinnvoll. Die monopolare Nadel sowie die Piezotechnik kamen ebenfalls nur selten zum Einsatz. Insbesondere der Piezoeinsatz bedeutet zusätzliche Kosten und ist am Knochen weniger effektiv als ein Bohrsystem.

Die sehr gute Eignung von Knorpel zur Rekonstruktion des Trommelfells und der hinteren Gehörgangswand sind hinlänglich bekannt [8]. Bei der Transplantatwahl der EES-Kliniken dominierte daher auch klar die Verwendung von Tragusknorpel mit Perichondrium und in geringerem Umfang Temporalisfaszie. Diese Materialien bieten sich bei Anlage eines endauralen Schnitts an, da beide ohne zusätzliche Inzision erreichbar sind. Insbesondere starres Transplantatmaterial wie Knorpel und Perichondrium lässt sich einhändig besser platzieren als die häufig feucht verwendete und dann flexible Temporalisfaszie. Conchaknorpel oder Xenografts spielten zur Rekonstruktion eine deutlich geringere bis gar keine Rolle.

Der Endoskopdurchmesser limitiert ohne Zweifel die Einsetzbarkeit dieses Instruments, weshalb dieser in einer Befragung bezüglich der EES stets mit erfragt werden sollte. In einer Studie an 201 computertomographischen Felsenbeinaufnahmen von Kindern und Heranwachsenden wurden für die Altersklassen von 1–3, 4–7, 8–11 und 12–18 Jahren die durchschnittlichen Gehörgangsdurchmesser und -flächen berechnet und in Beziehung zur Durchführbarkeit endoskopischer Operationen gesetzt. Die Autoren verwendeten ein Endoskop mit einem Optikdurchmesser von 2,7 mm. Hierbei wurde gezeigt, dass der für die EES-Technik erforderliche Durchmesser des Gehörgangs mindestens 5,1 mm betragen sollte, welcher meist bei einem Alter ab 8 Jahren erreicht wird [28].

In der vorliegenden Studie kamen passend zu dieser Publikation in 74 % der EES-Klinken Endoskope mit einem Durchmesser kleiner als 3 mm und mit einem Arbeitswinkel von 0°, 30° und 45° zur Anwendung. Die unterschiedlich eingesetzten Arbeitswinkel der Optiken entsprachen sicher den individuellen Anforderungen der jeweiligen Operation. Größere Durchmesser als 3 mm sind daher als Nachteil anzusehen, da sie die Bewegungsfreiheit v. a. bei Kindern einschränken und das chirurgische Vorgehen damit erschweren würden [20]. Die 3‑D-Technik scheint bei der EES in Deutschland bisher keine Rolle zu spielen.

Das Mikroskop halten 2 Drittel aller EES-Kliniken steril bezogen bereit. Bei diesen EES-Anwendenden wird offensichtlich mit einer hohen Wahrscheinlichkeit der Einsatz des Mikroskops erwartet. Zur Vermeidung eines intraoperativen Zeitverlusts ist daher eine entsprechende sterile Abdeckung des Endoskops und des Mikroskops notwendig. Der rein materielle Mehraufwand der sterilen Mikroskopabdeckung ist hierbei gegenüber einem möglichen Zeitverlust durch das intraoperative Beziehen zu vernachlässigen.

Indikationen für einen intraoperativen Wechsel von der EES zur mikroskopischen Technik sehen die meisten Kliniken bei Blutungen, erforderlichen bimanuellen Tätigkeiten oder bei umfangreicheren Bohrarbeiten am Mastoid. All diese Angaben entsprechen den Einschätzungen in der Literatur und sind mit einer schlechteren Sicht bei Bohrarbeiten und bei Blutungen verbunden [1, 22]. Weitere Indikationen für den Wechsel zum Mikroskop waren das Vorliegen einer Bogengangsfistel oder eines freiliegenden N. facialis. Möglicherweise sind diese Angaben auf das Erfordernis des beidhändigen mikroskopischen Arbeitens zurückzuführen und sind somit objektiv nachvollziehbar.

Die überwiegende Mehrheit der EES-Kliniken gab an, mehr als die Hälfte des Eingriffs mikroskopisch durchzuführen und lediglich endoskopisch zu flankieren. Rein endoskopische Eingriffe am Ohr wurden nur von einer Klinik angegeben.

An EES-Kliniken dominierten zu erwartende Einsatzorte, die mikroskopisch schlechter einsehbar sind. Das Protympanon sahen allerdings mit 49 % nur knapp die Hälfte der EES-Kliniken als geeignet an. Insbesondere dieser Bereich spielt sowohl bei der Ventilation des Mittelohrs als auch zur Kontrolle bei einer Cholesteatomausdehnung in die Tuba Eustachii eine Rolle [9]. Gerade EES-Anwendende sahen zusätzlich Arbeiten an vorderen Trommelabschnitten und Gehörgang als Stärke dieser Technik an, da vermutlich knöcherne Gehörgangserweiterungen zur Kontrolle dieser Abschnitte entfallen.

Als größter Nachteil der EES-Technik wurde das einhändige Operieren und die schlechtere Übersicht im Vergleich zur MES angegeben. Ohne Zweifel ist die Einhändigkeit für reine MES-Operateur*innen ungewohnt und bedarf eines Trainings. Die Einschätzung der Antwortenden einer schlechteren Übersicht bei der EES steht der physikalischen Eigenschaft eines größeren Bildausschnitts des Endoskops gegenüber. Gerade die Übersicht und die Einsicht in mikroskopisch verborgene Regionen ist als großer Vorteil des Endoskops anzusehen [15, 16, 23].

Entscheidend für die Etablierung und Weiterentwicklung einer Operationsmethode ist die Einschätzung des perspektivischen Stellenwerts. Lediglich 5 der 45 Kliniken schätzen den zukünftigen Stellenwert als „hoch“ oder „sehr hoch“ ein. Bei getrennter Betrachtung der übrigen Kliniken gaben alle Universitätsstandorte dies als mittel bis gering oder unklar an. Ähnlich schätzten dies die nichtuniversitären Kliniken ein, jedoch gab es hier zusätzlich 4 Standorte, die den Stellenwert zukünftig als sehr hoch in der Mittelohrchirurgie ansahen. Hiervon erfolgten an einer Klinik mehr als 20 und an zweien mehr als 50 % aller Mittelohreingriffe endoskopisch. Es existieren also durchaus Unterschiede in der perspektivischen Einschätzung zur zukünftigen Rolle der EES-Technik.

Es lässt sich somit zusammenfassend feststellen, dass sich seit der Erscheinung der ersten deutschsprachigen Übersichtsarbeit im Jahr 2016 [25] durchaus viel in Deutschland bezüglich des Einsatzes der EES-Technik verändert hat. Dies beruht offensichtlich in erster Linie auf der Implementierung dieser Operationsmethode an Universitätskliniken, aber auch nichtuniversitäre Einrichtungen sind sehr aktiv beteiligt. Insbesondere die aktuellere Literatur und der steile Anstieg der Anzahl an internationalen Publikationen der letzten 5 Jahre zu diesem Thema unterstreicht das hohe Potenzial der endoskopischen Ohrchirurgie [7, 21, 23, 24, 31, 37]. Die hier vorstellte Fragebogenerhebung hatte das ausschließliche Ziel, einen Überblick über den aktuellen Einsatz der EES in Deutschland zu geben. Dabei ist festzustellen, dass die endoskopische Chirurgie vielerorts ein Bestandteil der Ohrchirurgie in Deutschland geworden ist. Im internationalen Vergleich jedoch wäre die intensivere Anwendung dieser Technik anzustreben. Aus Sicht der Autoren könnte dies durch spezielle Fort- und Weiterbildungen [10], durch die weitere Standardisierung der chirurgischen und anatomischen Nomenklatur und v. a. durch die Entwicklung neuer Instrumente, wie beispielsweise eines Bohrsystems, erreicht werden. Keinesfalls kann die EES-Technik aktuell das Mikroskop voll ersetzen, sie ermöglicht allerdings ohne Zweifel neue Blickwinkel in der Ohrchirurgie.

## Limitationen

Zur Erhöhung der Aussagekraft dieser Umfrage wären folgende Inhalte sinnvoll:

- Zweiterhebung nach 5 Jahren
- Erweiterung des Fragenkatalogs durch folgende Gesichtspunkte:
  - Alter der EES-Anwendenden
  - Präzisierung der Indikationen am Gehörgang: Bei welchen Fragestellungen wird endoskopisch operiert?
  - Management intraoperativer Blutungen: Verwendung von Vasokonstringenzien, bipolare Kaustik, andere blutstillende Materialien?
  - Vorgehen bei Ossikuloplastik: Werden Autoossikel oder Prothesen verwendet? Werden die Rekonstruktionen stabilisiert? Mit welchen Instrumenten wird die Ossikuloplastik einhändig durchgeführt?
  - Besuch einer speziellen Fort- oder Weiterbildung?
  - Stellenwert der Entwicklung neuer Instrumente

## Ausblick

Die Umfrageergebnisse konnten zeigen, dass die EES-Technik in Deutschland unterschiedlich verbreitet ist und meist nur in Kombination mit der mikroskopischen Technik eingesetzt wird. Die Indikationen und praktische Durchführung der EES-Kliniken in Deutschland entsprechen weitestgehend den in der Literatur angegebenen Daten. Den zahlreichen internationalen Publikationen der letzten Jahre ist zu entnehmen, dass die zusätzlichen Möglichkeiten durch die EES den Horizont der Ohrchirurg*innen deutlich erweitern könnte. Dies sollte durchaus in Deutschland zu einer intensiveren Nutzung der EES führen. Vor diesem Hintergrund könnten verschiedene Maßnahmen, wie beispielsweise intensives, spezielles Training des operierenden Personals und die Entwicklung zusätzlicher Instrumente (insbesondere eines Bohrsystems) zukünftig im Fokus stehen. Das Ziel wäre hierbei, die EES-Technik zu einem festen Bestandteil in der ohrchirurgischen Ausbildung in Deutschland als sinnvolle Ergänzung zur mikroskopischen Mittelohrchirurgie werden zu lassen.

## Fazit für die Praxis

- EES („endoscopic ear surgery“) ist international eine anerkannte Operationstechnik.
- Endoskopische Ohrchirurgie wird an den meisten Universitätskliniken, aber auch intensiv an nichtuniversitären Kliniken in Deutschland durchgeführt.
- Der Aufwand der EES wird meist als höher eingeschätzt, was wahrscheinlich auf die anfänglich längeren Op.-Zeiten zurückzuführen ist.
- Aktuell wird die EES-Technik überwiegend flankierend zur mikroskopischen Technik angewendet.
- Nur wenige Kliniken führen EES-Eingriffe in größeren Fallzahlen durch.
- Im internationalen Vergleich entstand bei dieser Umfrage der Eindruck, dass die EES in Deutschland immer noch zurückhaltend eingesetzt wird.
- Eine spezielle Fort- und Weiterbildung sowie zusätzliche, speziell für die EES entwickelte Op.-Instrumente könnten die Anwendung breiter zugänglich machen.
- EES könnte als fester Bestandteil der Mittelohrchirurgie das Spektrum sinnvoll ergänzen und erweitern.
